# Interplay between energy metabolism and NADPH oxidase-mediated pathophysiology in cardiovascular diseases

**DOI:** 10.3389/fphar.2024.1503824

**Published:** 2025-01-10

**Authors:** Haipeng Jie, Jingjing Zhang, Shuzhen Wu, Luyao Yu, Shengnan Li, Bo Dong, Feng Yan

**Affiliations:** ^1^ Department of Cardiology, Shandong Provincial Hospital, Cheeloo College of Medicine, Shandong University, Jinan, China; ^2^ Department of Emergency Medicine, Qilu Hospital, Cheeloo College of Medicine, Shandong University, Jinan, China

**Keywords:** NADPH oxidases, oxidative stress, energy metabolism, cardiovascular diseases, ferroptosis

## Abstract

Sustained production of reactive oxygen species (ROS) and an imbalance in the antioxidant system have been implicated in the development of cardiovascular diseases (CVD), especially when combined with diabetes, hypercholesterolemia, and other metabolic disorders. Among them, NADPH oxidases (NOX), including NOX1-5, are major sources of ROS that mediate redox signaling in both physiological and pathological processes, including fibrosis, hypertrophy, and remodeling. Recent studies have demonstrated that mitochondria produce more proteins and energy in response to adverse stress, corresponding with an increase in superoxide radical anions. Novel NOX4-mediated modulatory mechanisms are considered crucial for maintaining energy metabolism homeostasis during pathological states. In this review, we integrate the latest data to elaborate on the interactions between oxidative stress and energy metabolism in various CVD, aiming to elucidate the higher incidence of CVD in individuals with metabolic disorders. Furthermore, the correlations between NOX and ferroptosis, based on energy metabolism, are preliminarily discussed. Further discoveries of these mechanisms might promote the development of novel therapeutic drugs targeting NOX and their crosstalk with energy metabolism, potentially offering efficient management strategies for CVD.

## 1 Introduction

Cardiovascular diseases (CVD) constitute a group of preventable conditions, including coronary heart disease, hypertension, heart failure, myocardial infarction, hypertrophic cardiomyopathy, and others. Since 1990, CVD has been the leading cause of death worldwide (Thomas et al., 2018). These diseases are associated with risk factors such as unhealthy diet, obesity, age, genetics, smoking, and diabetes (Schloss et al., 2020). The heart functions as the body’s pumping organ, driving blood circulation and requiring balanced energy metabolism to maintain its proper function (Bertero and Maack, 2018).

Cardiac energy metabolism is intricately linked with various metabolic substrates used by the heart. In a healthy heart, ATP is primarily produced through the oxidation of fatty acids, glucose, and lactate, whereas the fetal and newborn heart predominantly relies on glycolysis and lactic acid metabolism. As the heart matures, fatty acids become the preferred substrate (Kolwicz and Tian, 2011; Li et al., 2023). After birth, with changes in the levels of these substrates in the blood, the pattern of energy supplementation shifts, and the proportion of fatty acid oxidation (FAO) increases in healthy hearts (Li et al., 2023). Additionally, glucose, lactate, pyruvate, ketone bodies, and amino acids are also utilized as energy substrates. However, under various pathological conditions such as hypoxia, dyslipidemia, and diabetes, disturbances in energy metabolism and the development of CVD are interrelated (Li et al., 2023; Lopaschuk et al., 2021; Olkowicz et al., 2021), potentially mediated by NADPH oxidase (NOX) (Nabeebaccus et al., 2023; van der Pol et al., 2019; Zhang et al., 2020).

The NOX family comprises seven members in the human body, including NOX1-5 and DUOX1-2, all of which are multi-transmembrane proteins (NOX1-5 are six-pass transmembrane proteins, while DUOX1-2 are seven-pass transmembrane proteins) (Lambeth, 2004). All NOX enzymes share structural properties, including an NH2-terminal hydrophobic transmembrane region and a COOH-terminal flavin adenine dinucleotide binding domain. NOX1-4 include the catalytic subunit gp91phox (known as NOX2 in its most studied form), along with regulatory subunits p22phox, p47phox, p40phox, p67phox, and the small GTPase RAC. NOX5, based on gp91phox, has an amino-terminal calmodulin-like domain bound with calcium. DUOX1-2, based on NOX5, extend an amino-terminal peroxidase-homology domain (Begum et al., 2022; Gu et al., 2021). NOX enzymes are ubiquitously expressed in various subcellular localizations within cardiovascular tissues, regulating their physiological and pathological functions (Begum et al., 2022; Zhang et al., 2020).

Reactive oxygen species (ROS) are a chemically defined group that includes superoxide (O_2_•¯), hydrogen peroxide (H_2_O_2_), hydroxyl radical (OH•), and their reaction products, which can trigger cellular dysfunction. In the cardiovascular system, ROS originate from the mitochondrial electron transport chain, NOX, xanthine oxidase, lipoxygenase, and cyclooxygenase (Begum et al., 2022; Lambeth, 2004). Among these, NOX, as membrane protein donors, can be selectively activated to participate in physiological and pathological processes such as proliferation, migration, angiogenesis, and cell death (Begum et al., 2022). Based on NOX-mediated oxidative stress, this paper primarily focuses on the role of NOX-mediated energy metabolism in various CVD.

## 2 Cardiac energy metabolism

### 2.1 Fatty acid metabolism

The heart predominantly utilizes non-esterified fatty acids, chylomicrons, and very low-density lipoproteins for myocardial energy production (Hahn et al., 2023). Cardiomyocytes acquire fatty acids from plasma albumin (circulating) and lipoproteins for esterification. Fatty acid-binding protein on cardiomyocyte surfaces facilitates the uptake of free fatty acids (FFAs), which are transported into cardiomyocytes and recognized as substrates for fatty acid acetyl-coenzyme A synthetase, promoting lipid synthesis. These fatty acids also generate acetyl-CoA, which participates in the tricarboxylic acid (TCA) cycle to supply energy for the heart (Da Dalt et al., 2023; Gibb and Hill, 2018). High-fat diets, such as those rich in palmitic acid, can alter the heart’s fatty acid composition and slightly affect heart function in mice (Pakiet et al., 2020). Excessive accumulation of lipids in the heart can lead to oxidative and mitochondrial stress and apoptosis, partially modulated by NOX (Li et al., 2023; Nabeebaccus et al., 2023; Yamamoto and Sano, 2022).

### 2.2 Glucose metabolism

Glucose, another vital energy substrate for the heart, also provides metabolic substrates for various cell physiological activities (Kolwicz and Tian, 2011). Cardiomyocytes primarily take up circulating glucose via glucose transport proteins (GLUT), with GLUT1 (insulin-independent) and GLUT4 (insulin-dependent) as the main subtypes expressed in the heart (Aerni-Flessner et al., 2012). Glucose is then phosphorylated to glucose-6-phosphate (G-6-P) by hexokinase, producing pyruvate through glycolysis, which is transferred to the mitochondria to enter the TCA cycle for energy production. Additionally, glucose is utilized in the pentose phosphate pathway (PPP), glycogen synthesis, and the hexosamine biosynthetic pathway (HBP) (Aerni-Flessner et al., 2012; Gibb and Hill, 2018). The oxidative PPP is recognized for maintaining reduced glutathione levels to combat ROS by producing NADPH.

### 2.3 Lactate metabolism

Lactate is one of the sources of energy metabolism for the myocardium (Gibb and Hill, 2018). Before and after birth, the myocardium preferentially selects fatty acids over carbohydrates as energy substrates, a process accompanied by changes in lactate metabolism. Specifically, cardiomyocytes can take up and release lactate simultaneously, where lactate oxidation is inhibited by FAO (Bartelds et al., 1999). Typically, lactate provides about 10% of the energy supply required for daily consumption in the heart (Ouyang et al., 2023). Lactate and pyruvate are mutually converted to one another and can be produced or eliminated through a reversible redox reaction catalyzed by lactate dehydrogenase (Bonen, 2000). Monocarboxylate transporters (MCT) facilitate the transport of lactate from the blood into cardiomyocytes for lactate removal. Lactate is then oxidized to pyruvate, which subsequently enters the TCA cycle to provide energy for the heart. Interestingly, lactate has been demonstrated to enable the lactylation of lysine residues on histones or other proteins, playing protective roles in myocardial infarction (MI) and heart failure (Ouyang et al., 2023; Wang N. et al., 2022; Zhang et al., 2023).

### 2.4 Ketone body metabolism

Circulating ketone bodies, including acetoacetic acid, acetone, and β-hydroxybutyrate, serve as additional energy sources for the heart (de Koning et al., 2021; Manolis et al., 2023). These ketone body levels are increased in CVD such as heart failure, MI, and atherosclerosis, contributing to compensatory energy mechanisms (de Koning et al., 2021). Specifically, ketone bodies are produced by FAO in mitochondria and transported across cell membranes. Monocarboxylate transporters 1 and 2 (MCT1 and MCT2) facilitate their transport into cardiomyocyte mitochondria for ketolysis, thereby producing acetyl-CoA to enter the TCA cycle and produce ATP.

### 2.5 Branched-chain amino acid metabolism

Amino acids, including branched-chain amino acids (BCAAs: valine, leucine, and isoleucine), glutamic acid, cystine, histidine, and lysine, serve as a fuel source for the heart (Lopaschuk and Ussher, 2016). The metabolism of amino acids is closely related to obesity, ischemic cardiomyopathy, heart failure, and other diseases, and may predict the risk of cardiovascular events (McGarrah and White, 2023; Shah et al., 2012). Specifically, BCAAs are first transaminated by branched-chain amino acid transferases (BCATs) to form branched-chain α-keto acids (BCKAs) and glutamate (Dimou et al., 2022). Subsequently, BCKAs are oxidized and decarboxylated to their branched-chain acyl-CoA esters, with α-ketoglutarate dehydrogenase playing a pivotal role (Patrick et al., 2022). Finally, various branched-chain acyl-CoA compounds promote cardiac energy production through different pathways.

## 3 The role of ROS in physiological processes

Most studies indicate that excessive ROS accumulation leads to DNA damage, protein modification, and lipid peroxidation, ultimately inducing irreparable cellular injury (Checa and Aran, 2020). However, it is noteworthy that localized, low levels of ROS are critical for redox signaling. Current research predominantly focuses on H₂O₂, which functions as a second messenger in signaling pathways. H₂O₂ mediates protein post-translational modifications (e.g., cysteine and tyrosine residues), transcription factor activity, and epigenetic modifications of DNA and histones (Lennicke and Cochemé, 2021a). These processes regulate downstream signaling pathways involved in autophagy, cell proliferation, apoptosis, extracellular matrix repair, and immune defense (Checa and Aran, 2020; Holmström and Finkel, 2014). While the precise mechanisms are not detailed here, we emphasize the relationships between ROS and energy metabolism under physiological conditions. Generally, ROS modulates cellular energy metabolism by activating or inhibiting protein kinases and suppressing phosphatases. This is evident in signaling pathways such as insulin, AMPK, and mTOR (Checa and Aran, 2020; Lennicke and Cochemé, 2021a; Zmijewski et al., 2010). In the insulin signaling pathway, ligand-receptor interactions induce ROS production via NOX4, which is critical for proper cascade reaction and glucose metabolism (Lennicke and Cochemé, 2021b). Interestingly, NOX4 has been shown to improve high-fat diet-induced adipose accumulation, insulin resistance, and liver steatosis (Li et al., 2012). Notably, NOX4 produces H₂O₂ instead of O_2_•¯, which may play vital roles through redox signaling (Schürmann et al., 2015). AMPK is activated when energy metabolism demand increases, and studies have shown that H₂O₂ can activate AMPK by oxidizing its cysteine residues (Zmijewski et al., 2010). In mitochondria, ROS is the byproduct of the respiratory chain, linking them closely to cellular metabolism (Shadel and Horvath, 2015). Recent findings suggest that elevated hypothalamic ROS suppresses food intake and increases energy expenditure, indicating the physiological roles of ROS in maintaining energy homeostasis (Benani et al., 2007). While most studies focus on the pathological overproduction of ROS driven by NOX, their roles in physiological processes, particularly in relation to cellular metabolism, remain underexplored and warrant further investigation.

## 4 The roles of NOX in CVD

### 4.1 Atherosclerosis

Atherosclerosis is a pathological condition characterized as a chronic multifocal immune-inflammatory disease driven by lipids, mainly occurring in large and medium-sized arteries (Tedgui and Mallat, 2006). NOX1, NOX2, NOX4, NOX5 are the main source of ROS in vasculature (Langbein et al., 2016). Upregulated NOX1 in atherosclerosis has been demonstrated to promote vascular smooth muscle cell (VSMC) proliferation and extracellular matrix (ECM) production, inducing the formation of vascular neointima (Lee et al., 2009; Valente et al., 2012). Knockout NOX1 has been shown to delay the progression of atherosclerosis by reducing ROS production, suppressing inflammation, improving mitochondrial apoptosis, and alleviating endothelial cell dysfunction (Liu et al., 2020; Sorescu et al., 2004). NOXO1 (NOX organizer 1) and NOXA1 (NOX activator 1) are necessary for the activation of NOX1 and their inhibition produces similar effects (Buchmann et al., 2020; Siu etal., 2016). In atherosclerosis, NOX1 is elevated early but declines later, while NOX4 increases in advanced stages (Xu et al., 2014). Unlike the harmful effects of other NOX isoforms, the roles of NOX4 in atherosclerosis remain under discussion. Recent studies have revealed that NOX4-derived H₂O₂ has vascular protective effects (Langbein et al., 2016; Schürmann et al., 2015). At the same time, overexpression of NOX4 in endothelial cells reduces the expression of interferon-gamma and increases the proportion of T regulatory cells (Craige et al., 2015). However, the proinflammatory phenotype of VSMCs mediated by NOX4 is also recognized to cause plaque instability and rupture (Xu et al., 2014). Recent studies have revealed that the overexpression of NOX4 in mitochondria can accelerate the formation of aortic sclerosis. This effect can be partially reversed by mitochondrial oxidative stress inhibitors (Canugovi et al., 2019; Vendrov et al., 2015).

The unique role of NOX4 in atherosclerosis may be linked to its production of H₂O₂, which functions as a second messenger widely involved in regulating cellular signaling pathways. However, the precise mechanisms remain unclear. Beyond its role in modulating inflammation infiltration and VSMC phenotype switching, the metabolic effects of NOX4 warrant further investigation. The current study has shown that silencing NOX4 in hepatocytes reduces insulin and fatty acid utilization, but its metabolic role in atherosclerosis remains to be elucidated (Wu and Williams, 2012).

### 4.2 Hypertension

Essential hypertension, a major contributor to the global disease burden, is closely associated with a high-salt diet, obesity, dyslipidemia, and diabetes (Di Raimondo et al., 2021; Munir and du Toit, 2024; Poulter et al., 2015). NOX1 and NOX4 are significantly elevated in VSMC of spontaneously hypertensive rats (SHR), particularly in the endoplasmic reticulum (ER) and nucleus, promoting ROS production and ER stress through protein sulfenylation and hyperoxidation (Camargo et al., 2018). Moreover, associations between NOX and hypertension have been demonstrated in animal models induced by L-NAME, Ang-II, and DOCA-salt, affecting both the vascular system and extravascular systems, including the renal system, central nervous system, and immune system (Griendling et al., 2021; Zhang et al., 2020). Several studies have revealed the harmful roles of NOX1/2-mediated oxidative stress in hypertension, including but not limited to the inactivation of NO and the production of peroxynitrite (Drummond et al., 2011; Drummond and Sobey, 2014; Matsuno et al., 2005; Murdoch et al., 2011). However, NOX4 remains controversial in the pathologic progression of hypertension. Some studies have demonstrated that NOX4 has similar effects to other NOX, but recent studies have revealed that NOX4 plays a protective role in vasculature by producing H₂O₂ and NO (Drummond et al., 2011; Ray et al., 2011; Schröder et al., 2012). Specially, this includes reducing Ang-II induced vascular dysfunction and increasing ischemia induced angiogenesis (Schröder et al., 2012).

Recently, NOX4-mediated metabolism remodeling has gradually come into view (Nabeebaccus et al., 2023). In Dahl salt-sensitive (SS) hypertensive rats, kidney metabolomics reveal that the PPP and glycolysis are increased, accompanied by reduced glutathione reductase activity and TCA cycle activity, promoting ROS mediated by NOX (Wang Y. et al., 2018). Additionally, fatty acid and amino acid metabolism also change (Wang Y. et al., 2018). Recent clinical studies reveal that high salt intake in daily life increases serum sodium and osmotic pressure, which are considered risks for hypertension (Kuwabara et al., 2020). It is also demonstrated that sodium and fructose intake contribute to metabolic syndrome, consisting of insulin resistance, obesity, dyslipidemia, and hypertension in children (Genovesi et al., 2021). Fructose, whether produced by the liver on a high-salt diet or ingested externally, can increase intracellular uric acid levels. This, in turn, recruits NOX into mitochondria, leading to oxidative phosphorylation uncoupling and oxidative stress, ultimately contributing to hypertension (Sánchez-Lozada et al., 2023). Interestingly, knocking out NOX4 reduces the utilization of glucose and lipids, inducing insulin signaling disturbances (Lennicke and Cochemé, 2021b; Li et al., 2012; Wu and Williams, 2012). Therefore, NOX4-mediated metabolic remodeling may also be involved in hypertension.

**FIGURE 1:**
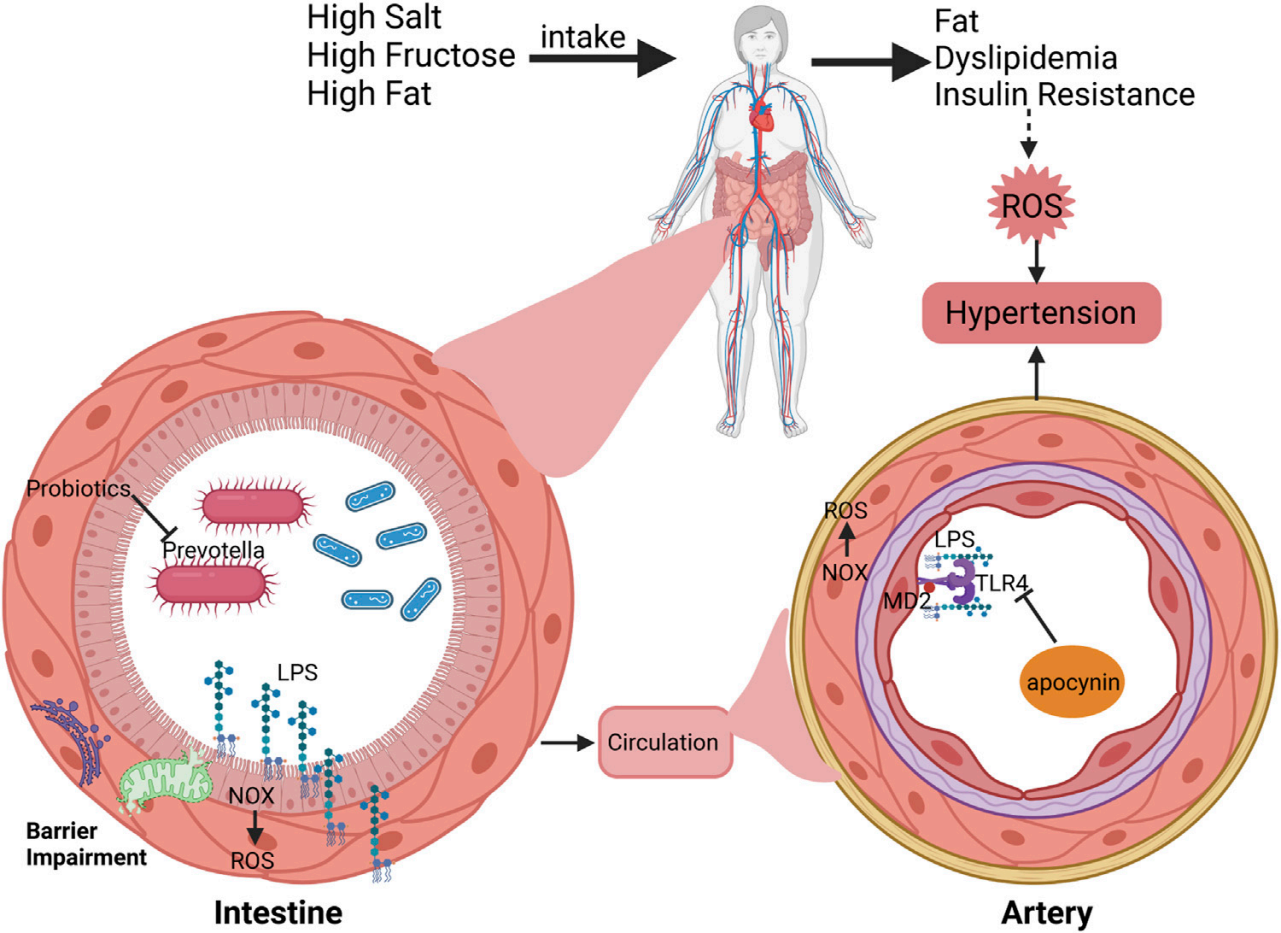
Interactions between hypertension, dietary factors, gut microbiome, and NOX pathways. Daily intake of high levels of salt, fructose, and fat could cause obesity, dyslipidemia, insulin resistance and hypertension. In particular, changes in daily diet induce dysfunction of gut microbiome and NOX-mediated ROS production, resulting in barrier damage and chronic LPS translocation to the circulation. And LPS also acts on the arterial endothelium through the TLR4/MD2 complex, promoting oxidative stress to cause hypertension.

Given the predominant influence of diet on hypertension, the gut microbiome in hypertension patients has attracted significant interest. Studies have revealed that Prevotella is a main contributor to prehypertension and hypertension, and mice that receive stool from hypertensive patients experience elevated blood pressure (Li et al., 2017). Fecal microbiota transplantation from losartan-treated SHR improves endothelial function and reduces NOX activity in untreated SHR, leading to lower blood pressure (Robles-Vera et al., 2020). Dysregulation of the gut microbiota and barrier impairment during hypertension induces chronic translocation of lipopolysaccharide (LPS) into circulation, causing an imbalance of NOX and antioxidant enzymes through toll-like receptor 4 (TLR4). This finding provides a theoretical basis for the use of probiotics in treating hypertension (Grylls et al., 2021; Kim et al., 2018; Robles-Vera et al., 2020). In response to high-fat diets, TLR4 knockout prevents obesity-induced endothelial dysfunction and hypertension by reducing NOX1/4 and ROS content, lowering inflammatory factors, and increasing eNOS activity (Liang et al., 2013). Blocking the TLR4-MD2 complex effectively improves blood pressure by reducing oxidative stress (de Oliveira et al., 2020). Additionally, apocynin, a selective NOX inhibitor, reduces the stability and expression of LPS-induced TLR4, indicating the crosstalk between oxidative stress produced by NOX p47^*phox*^ and TLR4 or its subsequent pathways (Lin et al., 2006). Similarly, decreased expression of NOX1 and NOX4 in the vascular tissue of TLR4-mutated mice protects against arterial endothelial dysfunction in diabetic mice (Liang et al., 2013) (Figure 1).

Long-term intake of a high-fat, high-sugar diet increases vascular wall oxidative stress through enteral (gut microbiota) and extrenteral pathways, causing vascular remodeling and hypertension. NOX4 may play a vascular protective role due to the production of H_2_O_2_ and NO. At the same time, H_2_O_2_, as a second messenger, may also participate in downstream signaling pathways, but further confirmation is still needed.

### 4.3 Heart failure

Heart failure (HF) is a decompensated manifestation of various CVD, seriously threatening the health and quality of life of patients (Ziaeian and Fonarow, 2016). The imbalance between oxidative stress and endogenous antioxidant systems accelerates the pathogenesis of heart failure (van der Pol et al., 2019; Zhang et al., 2020). A failing heart exhibits significant changes in energy metabolism, accompanied by reduced ATP synthesis, and shows a preference for ketone body and lactate metabolism over FAO (Lopaschuk et al., 2021; Montaigne et al., 2021). Myocardial metabolomics reveal that amino acids (especially BCAAs), pyruvate, and ketone bodies are increased in patients with heart failure, acting as supplementary substrates in the TCA cycle to maintain energy homeostasis (Hahn et al., 2023). Mitochondrial oxidative phosphorylation is essential for ATP production, and functional impairment in this process is frequently accompanied by the production of superoxide anions, especially in the energy-intensive heart. Therefore, exploring the crosstalk between oxidative stress mediated by NOX and metabolism remodeling in heart failure is crucial. Multiple studies have revealed that NOX2, NOX4, and NOX5 increase ROS production, promoting cardiac pathological remodeling and dysfunction (Lozhkin et al., 2022; Parajuli et al., 2014; Zhao et al., 2020). Specifically, knockdown of NOX2 improves oxidative stress, myocardial fibrosis, and remodeling in the transverse aortic constriction (TAC) model, while also inhibiting MAPK activation (Parajuli et al., 2014). ROS have been demonstrated to activate MAPK, inducing the differentiation of fibroblasts into myofibroblasts and the production of inflammatory factors (Romero-Becerra et al., 2020). Moreover, NOX4 promotes the differentiation of fibroblasts into myofibroblasts through the TGF-β/SMAD pathway (Cucoranu et al., 2005). Overexpression of NOX4 in mitochondria leads to mitochondrial dysfunction and fission, causing cardiac dysfunction mediated by oxidative stress. The possible mechanism involves a significant decrease in the activity of mitochondrial citrate synthase and complex I, leading to oxidative phosphorylation decoupling (Lozhkin et al., 2022). Interestingly, the increased expression of NOX4 in heart failure may be a compensatory effect. Recent studies have shown that NOX4 induces the expression and release of Hypoxia-inducible factor 1 alpha (HIF-1α) and vascular endothelial growth factor (VEGF), increasing myocardial capillary density to improve cardiac function (Zhang et al., 2010). NOX5, a calcium-sensitive subtype, has also been demonstrated to promote myocardial hypertrophy and systolic dysfunction (Zhao et al., 2020).

**FIGURE 2:**
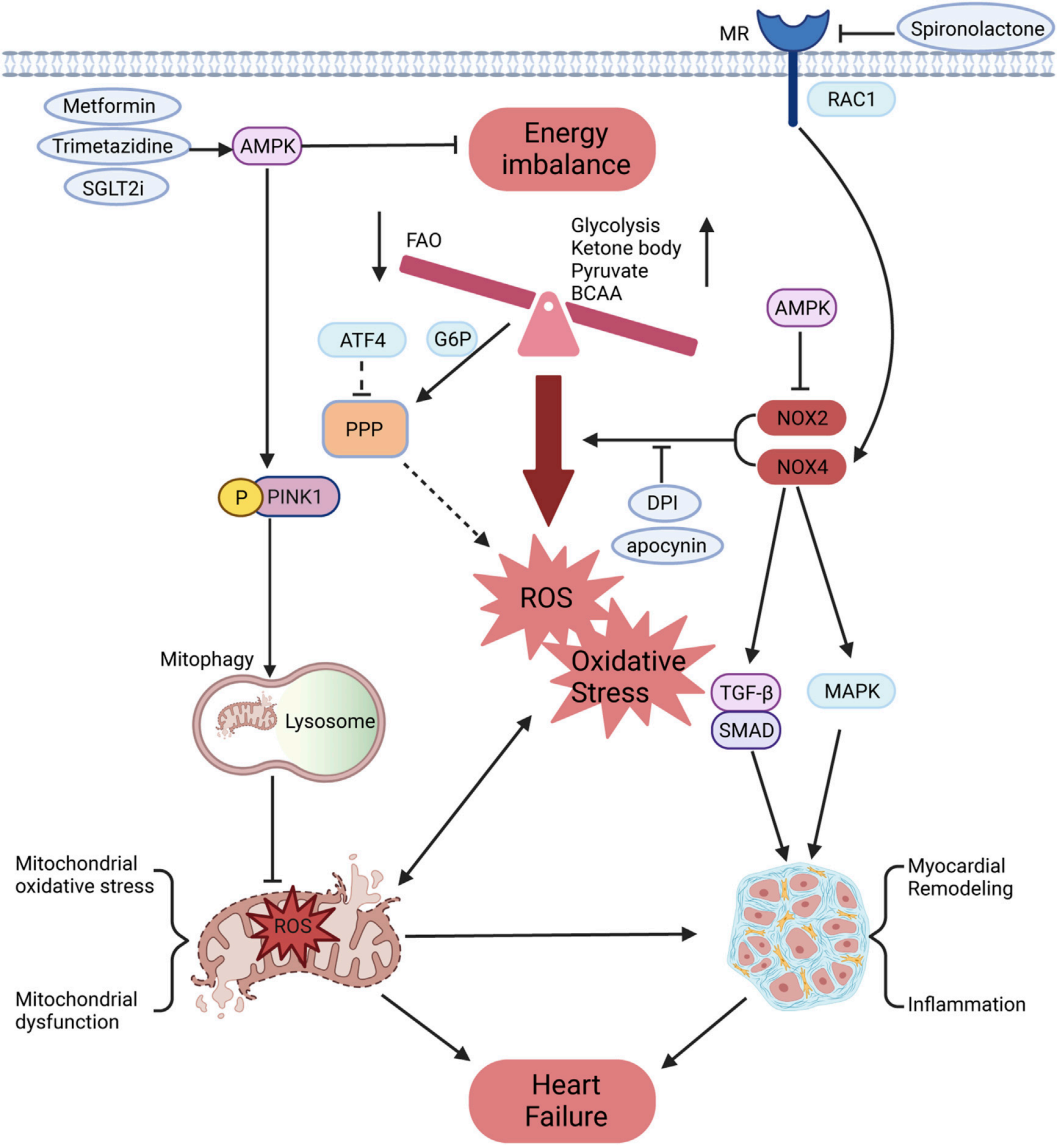
Effects of NOX on energy metabolism and oxidative stress in HF. The cardiac energy metabolism of HF is manifested as reduced FAO, increased metabolism of glucose, ketone bodies, pyruvate, BCAAs, and other substrates, which is accompanied by oxidative phosphorylation imbalance, leading to ROS production and oxidative stress. The PPP, a glycometabolic bypass, is also enhanced in HF and might provide more oxidizing substrates for NOX to promote ROS production. Meanwhile, NOX could promote the conversion of fibroblast into myofibroblast and increase the expression of inflammatory factors through TGF-β/SMAD and MAPK pathways, leading to myocardial remodeling. The mineralocorticoid receptor can be activated by RAC1 to promote NOX4 expression, which is partially inhibited by mineralocorticoid receptor antagonists. As the core regulator of energy homeostasis, AMPK can inhibit NOX activity and phosphorylate PINK1 to restore damaged mitophagy. This process improves mitochondrial function and reduces oxidative stress, thereby preventing the progression of HF.

Glucose-6-phosphate dehydrogenase (G6PD), the first regulatory enzyme in the PPP, is significantly upregulated in patients with HF, resulting in increased superoxide production, which can be suppressed by inhibitors of NOX and G6PD (Gupte et al., 2007). This phenomenon has been further demonstrated in HF models (Gupte et al., 2006). Previous studies have shown that glycolysis is upregulated in HF, potentially increasing the utilization of glycolytic intermediates and glucose to enhance PPP activity and induce ROS production (Bertero and Maack, 2018). Insulin resistance in ischemic heart failure activates NOX, which can be alleviated by enhancing Akt phosphorylation and GLUT4 translocation using apocynin (Fukushima et al., 2016; Ohta et al., 2011). However, the above studies did not identify specific types of NOX. Most studies have shown that NOX2 can produce oxidative stress that mediates insulin resistance (Souto Padron de Figueiredo et al., 2015; Zhang et al., 2014). Interestingly, NOX2 deficiency reduced the improvement of exercise training on diet-induced insulin resistance and obesity (Henriquez-Olguin et al., 2023). To some extent, NOX4 is universally recognized to improve insulin resistance due to producing H₂O₂, which activates insulin receptor kinase and inhibits phosphatase (Lennicke and Cochemé, 2021b; Li et al., 2012; Plecitá-Hlavatá et al., 2020). However, NOX4 has also been demonstrated to cause insulin resistance and inflammation in adipose tissue (Den Hartigh et al., 2017). Although there is substantial evidence that NOX4 can increase insulin signaling, further validation is needed. Increased glycolysis is not sufficient to compensate for inadequate energy metabolism in heart failure, whereas NOX4 may improve metabolic remodeling in pathological states to improve heart function (Lopaschuk et al., 2021). AMP-activated protein kinase (AMPK), a key energy sensor regulating glucose and lipid metabolism, is influenced by oxidative stress primarily derived from NOX to maintain cellular homeostasis (Song and Zou, 2012). Trimetazidine improves insulin resistance and mitochondrial function through AMPK activation, protecting against TAC-induced HF (Shu et al., 2021). Further studies reveal that the AMPKα2 subtype restores impaired mitophagy by phosphorylating PINK1 at Ser495, thereby enhancing mitochondrial function and reducing oxidative stress to mitigate HF progression (Wang B. et al., 2018). Therefore, AMPK activators such as metformin and statins can improve impaired heart function by reducing NOX activity and maintaining mitochondrial and energy metabolism homeostasis (Song and Zou, 2012). Mineralocorticoid receptor (MR) antagonists, including spironolactone and eplerenone, are fundamental drugs in HF management, as they partly inhibit NOX activity to reduce oxidative stress (Nagata et al., 2006). MR activation by small GTPase Rac1 increases NOX4 expression in TAC-induced HF (Ayuzawa et al., 2016). Sodium-glucose cotransporter 2 (SGLT2) inhibitors, which are new therapeutic drugs for HF, enhance the AMPK/Rac1 pathway to inhibit NOX activity and increase tetrahydrobiopterin bioavailability through additional SGLT1 inhibition effects (Heidenreich et al., 2022; Kondo et al., 2021). Moreover, ATF4 targets several enzymes in the PPP and one-carbon metabolic pathways to maintain redox homeostasis in the heart, findings validated by metabolomic and transcriptomic analyses (Wang X. et al., 2022) (Figure 2).

### 4.4 Myocardial infarction

Myocardial infarction (MI) is a severe event characterized by a sudden decrease in blood supply and energy deprivation, often accompanied by metabolic remodeling and oxidative stress (Li et al., 2023; Liu et al., 2022; Zhang et al., 2020). Metabolomic studies have shown significant changes in glucose, amino acid, and ketone metabolism in peripheral blood and tissues during MI (Bai et al., 2020; Liu et al., 2022). The role of NOX1 in accelerating vascular disease progression has been extensively studied, but its specific role in the heart remains a topic of ongoing investigation. In MI mice subjected to ischemia-reperfusion, knockdown of NOX1/NOX2 decreases the size of MI, although oxidative stress levels remain unchanged in NOX1-deficient mice (Braunersreuther et al., 2013). However, inhibition of NOX1 does not improve heart function in MI mice, and late ischemic preconditioning actually increases MI size and apoptosis, potentially by blocking protective effects mediated by NF-kB activation (Jiang et al., 2014). Knockdown of NOX2 or p47phox improves systolic and diastolic function in MI models, accompanied by reduced fibrosis and apoptosis (Doerries et al., 2007; Looi et al., 2008). Conversely, specific overexpression of NOX2 in cardiomyocytes or endothelium does not deteriorate systolic and diastolic function, although cardiomyocyte overexpression led to increased fibrosis and myocardial hypertrophy (Sirker et al., 2016). NOX2-specific siRNA and microRNA oligonucleotide particles containing miR-106b, miR-148b, and miR-204 have been designed to reduce oxidative stress and improve heart function through intramyocardial injection (Somasuntharam et al., 2013; Yang et al., 2017). In contrast to NOX2, cardiomyocyte overexpression of NOX4 promotes M2 macrophage polarization, improving myocardial remodeling and survival in MI mice, while also increasing autophagy to cope with energy stress (Mongue-Din et al., 2017; Sciarretta et al., 2013). However, inhibition of NOX4 reduces MI size, accompanied by decreased oxidative stress and inflammatory infiltration (Stevenson et al., 2019). Increased NOX4 expression in the paraventricular nucleus impairs heart function through oxidative stress-mediated activation of sympathetic nerves in MI mice, an effect that is mitigated by NOX4 inhibition in the paraventricular nucleus (Infanger et al., 2010). The roles of NOX1 and NOX4 in MI demonstrate contradictory effects that warrant further investigation (Hahn et al., 2012). There are fewer studies showing the associations between NOX5 and MI, which could cause endothelium dysfunction by regulating calcium or oxidase stress to exacerbate disease progression (Marqués et al., 2022; Zhao et al., 2020).

**FIGURE 3:**
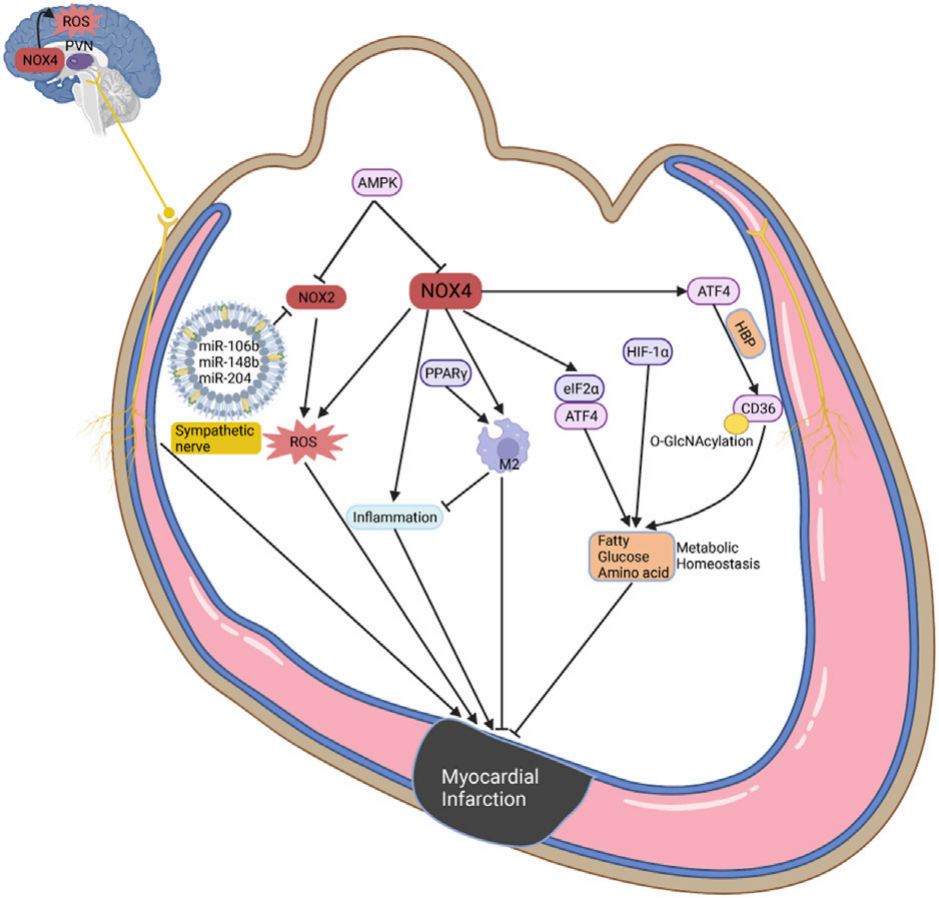
The potential roles of NOX in oxidative stress and metabolic homeostasis during MI. The most common function of NOX2/4 is to produce ROS and oxidative stress, which can be inhibited by AMPK. In the paraventricular nucleus, NOX promotes ROS production and activates sympathetic nerve to deteriorate cardiac function. And NOX4 can increase inflammatory infiltration, but is also recognized to promote macrophage conversion to M2 phenotype, and improves myocardial remodeling. Interestingly, NOX4 maintains metabolic homeostasis during MI through ATF4, eIF2α, and HIF-1α, including but not limited to fatty acid, glucose, and amino acid metabolism.

Previous studies have revealed that metabolic dysfunctions in glycolysis, FAO, ketone bodies, and BCAAs promote the progression of MI (Li et al., 2023). Under hypoxic conditions, cardiomyocytes preferentially utilize glycolysis for ATP production, increasing GLUT1/4 expression and enhancing the affinity of phosphofructokinase 1 for substrates, a process promoted by AMPK (Li et al., 2023; Sosa et al., 2007). Multiple studies have demonstrated that H₂O₂ produced by NOX4 promotes insulin signaling, which probably promotes metabolic balance (Lennicke and Cochemé, 2021b; Plecitá-Hlavatá et al., 2020). Metformin, an AMPK activator, has been shown to reduce oxidative stress mediated by NOX4 (Sosa et al., 2007). Overexpression of NOX4 in cardiomyocytes reduces glycolysis and promotes FAO by increasing O-linked N-acetylglucosamine (O-GlcNAcylation) and CD36 combination, regulated by ATF4 via the hexosamine biosynthetic pathway (Nabeebaccus et al., 2017). NOX4 also protects cell survival during MI through the eIF2α/ATF4 pathway, driving pro-survival and metabolic transcriptional programs including autophagy and amino acid metabolism (Nabeebaccus et al., 2023). HIF-1α translocates into the nucleus under hypoxic conditions to induce protective pathways such as increased glycolysis, enhanced lactate transportation, and stabilization of cellular homeostasis, which includes autophagy, mitophagy, and management of oxidative stress (Ong and Hausenloy, 2012; Sciarretta et al., 2013). Notably, NOX2/4 may serve as upstream and downstream regulators of HIF-1α, playing pivotal roles in oxidative stress during myocardial energy metabolism (Nabeebaccus et al., 2023). Exogenous fat supplementation can exacerbate oxidative stress during MI (Liu and Lloyd, 2013). The peroxisome proliferator-activated receptor (PPAR) family, including PPARα, PPARγ, and PPARδ, are crucial regulators of lipid metabolism in CVD (Montaigne et al., 2021). The role of PPARα in MI is still controversial. PPARα possibly improves insulin resistant and oxidative stress, but overexpression of PPARα in cardiomyocytes causes glycogen deposition, macrophage infiltration, antioxidant system imbalance, and heart function deterioration (Duerr et al., 2014; Ibarra-Lara et al., 2016; Wagner and Wagner, 2020). Despite multicenter randomized controlled trials showing that the PPARα agonist pemafibrate does not reduce cardiovascular events in patients with type 2 diabetes or dyslipidemia, its efficacy remains uncertain (Das Pradhan et al., 2022). Several studies have demonstrated that PPARγ is upregulated in MI and exerts protective effects. Increased myocardial inflammatory infiltration and NOX2/4 exacerbate heart function following PPARγ knockdown in myeloid cells (Shen et al., 2018; Wagner and Wagner, 2020). Similar to NOX4, the PPARγ agonist pioglitazone reduces macrophage infiltration and promotes macrophage conversion to the M2 phenotype, improving myocardial remodeling (Mongue-Din et al., 2017; Tokutome et al., 2019) (Figure 3).

### 4.5 Hypertrophic cardiomyopathy

Hypertrophic cardiomyopathy (HCM) is characterized by ventricular hypertrophy and abnormal load with unknown etiology, often presenting with myocardial fibrosis, cardiomyocyte hypertrophy, and disordered arrangement (Previs et al., 2022; Teekakirikul et al., 2019). Several proteomics and metabolomics studies reveal that the imbalance of energy metabolism in HCM not only involves decreased FAO, glycolysis and intermediates of TCA, but also increases the utilization of ketone body, lactate and BCAAs, eventually causing impaired ATP production. Oxidative stress, mitochondrial dysfunction and impaired mitophagy are also demonstrated in HCM, which mutually exacerbate metabolic disorder (Coats et al., 2018; Previs et al., 2022; Ranjbarvaziri et al., 2021). NOX inhibitor apocynin, mitochondria-targeted antioxidant SS-31, and mitoquidone are also proven to alleviate myocardial dysfunction, remodeling and fibrosis mediated by oxidative stress and mitochondrial dysfunction (Dai et al., 2011; Goh et al., 2019; Saleem et al., 2018). Mice with specific overexpression of NOX1 in VSMC develop vascular hypertrophy, which is associated with oxidative stress and can be prevented by the antioxidant tempol (Dikalova et al., 2005). In TAC-induced HCM model, NOX2 has been shown to promote oxidative stress, cardiac dysfunction, and fibrosis (Grieve et al., 2006; Parajuli et al., 2014). The role of NOX4 in HCM remains controversial, as studies have suggested both protective and detrimental effects. Similar to NOX2, NOX4 is generally recognized to enhance oxidative stress and contribute to cardiac dysfunction in HCM, primarily through pathways involving mTOR and NF-kB (Ago et al., 2010; Zhao et al., 2015). NOX4 siRNA delivered via small extracellular vesicles targeting the heart has shown potential in mitigating cardiac dysfunction and fibrosis in Ang-II-induced HCM models (Kang et al., 2023). Conversely, previous studies indicate that increased cardiac capillary density in NOX4-overexpressing HCM may provide protection against heart dysfunction and facilitate adaptation to chronic stress, possibly regulated by the HIF-1α/VEGF axis (Zhang et al., 2010). Specific knockdown of NOX4 in cardiomyocytes and endothelial cells has been shown to exacerbate myocardial dysfunction and remodeling in TAC models (Zhang et al., 2018). Additionally, NOX5 has been implicated in inducing heart dysfunction and fibrosis in HCM through oxidative stress mechanisms (Zhao et al., 2020).

**FIGURE 4:**
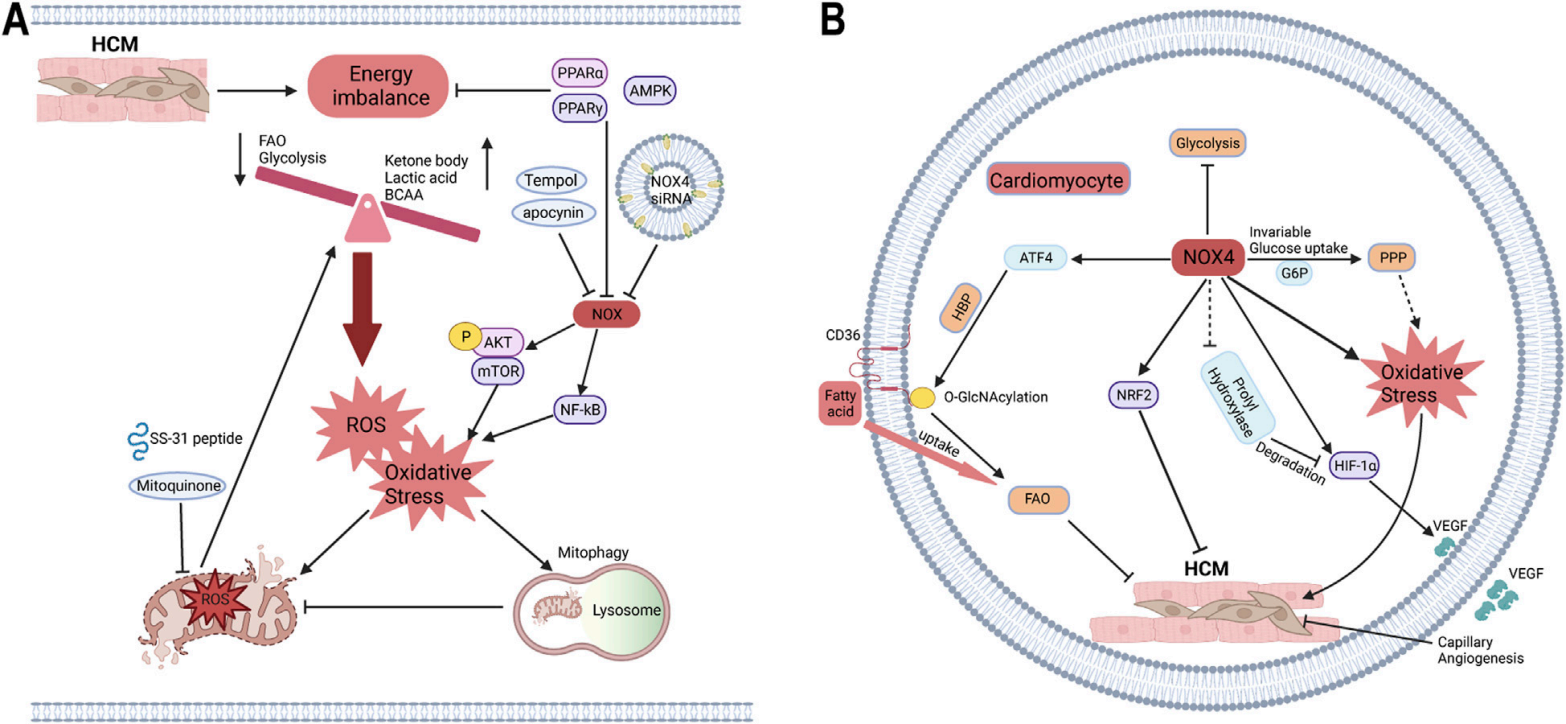
The roles of NOX in HCM in the context of energy metabolism and oxidative stress. **(A)** The crosstalk of NOX on energy metabolism and oxidative stress. The cardiac energy metabolism of HCM is manifested as decreased FAO and glycolysis, increased metabolism of ketone bodies, pyruvate, BCAAs, etc., accompanied by mitochondrial dysfunction and oxidative phosphorylation imbalance leading to ROS production and oxidative stress. Meanwhile, NOX can promote oxidative stress through p-AKT/mTOR and NF-kB pathways, and further induce mitochondrial oxidative stress and dysfunction, which are inhibited by apocynin, DPI, mitoquinone, mitochondria-targeted antioxidant SS-31, and NOX4 siRNA. PPARα, PPARγ, and AMPK can maintain energy homeostasis and inhibit NOX activity, which may be a bridge between energy metabolism and oxidative stress. **(B)** The paradoxical role of NOX4 in HCM. Traditionally, the most classic role of NOX4 is to produce oxidative stress. Overexpression of NOX4 does not change glucose uptake but inhibits glycosis, possibly providing oxidizing substrates via glycometabolic bypass PPP. And HBP, as a glycometabolic branch, increases the O-GlcNAcylation of CD36 to promote FAO. Meanwhile, NOX4 promotes myocardial capillary angiogenesis via HIF-1α/VEGF, possibly by inhibiting prolyl hydroxylase activity to reduce HIF-1α degradation. NOX4 also activates NRF2 to protect against HCM without capillary angiogenesis. FAO, fatty acid oxidation; PPP, pentose phosphate pathway; HBP, hexosamine biosynthesis pathway.

As described above, stress-overloaded hearts exhibit decreased FAO and glycolysis, accompanied by mitochondrial dysfunction and oxidative stress, leading to disturbances in energy supply. The balance between oxidative phosphorylation and oxidative stress is critical for maintaining cellular homeostasis. The contradictory role of NOX4 in HCM may be linked to simultaneous increases in FAO and oxidative stress, which can respectively be protective and harmful. Recent studies have shown that in hearts overexpressing NOX4, FAO is upregulated, whereas glucose oxidation is reduced, despite no significant change in glucose uptake. This metabolic shift is facilitated through the activation of the HBP, which enhances FAO by increasing CD36 expression (Nabeebaccus et al., 2017). PPP is also recognized as a glucose utilization branch and is enhanced by increased G6PD activity, leading to elavated superoxide production, which may have adverse effects in NOX4 overexpression (Gupte et al., 2007). Key regulators such as the PPAR family, NRF2, and AMPK play pivotal roles in energy metabolism and redox homeostasis under various stress conditions, including in HCM (Hayes and Dinkova-Kostova, 2014; Li et al., 2023; Nabeebaccus et al., 2023). Activators of PPARα and PPARγ have been shown to reduce the levels of p22phox and p47phox in endothelial cells, thereby aiding in redox balance (Inoue et al., 2001). Conversely, NOX2-mediated oxidative stress downregulates PPARα expression, exacerbating heart dysfunction in HCM (Harvey et al., 2020). The interplay between NOX enzymes and the PPAR family involves complex redox and metabolic crosstalk, which requires further investigation. Chronic oxidative stress mediated by NRF2 can induce myocardial remodeling, mitochondrial dysfunction, and caspase 3-independent cell death, ultimately leading to HCM and heart failure (Shanmugam et al., 2020; Smyrnias et al., 2015). Interestingly, NRF2 activation by endogenous NOX4 has shown a protective role in HCM, independent of increased capillary density (Smyrnias et al., 2015) (Figure 4).

### 4.6 Diabetic cardiomyopathy

Diabetic cardiomyopathy (DCM) is a form of heart dysfunction that occurs in the context of diabetes, without the presence of coronary artery disease (CAD), hypertension, or valvular diseases. It is characterized by myocardial insulin resistance, mitochondrial dysfunction, and abnormal coronary microcirculation (Jia et al., 2016). Unlike MI and HCM, DCM is marked by increased FAO and decreased glycolysis, leading to lipid toxicity and oxidative stress. These processes disrupt cellular metabolism and contribute to mitochondrial dysfunction (Jia et al., 2016; Kenny and Abel, 2019; Tong et al., 2019). Both redox imbalance and metabolic disorders play roles in the pathogenesis of DCM, although the crosstalk between these factors remains enigmatic (Kenny and Abel, 2019). Specifically, oxidative stress mediated by NOX1 exacerbates heart dysfunction and fibrosis through the TLR2/NF-κB pathway, a process that can be ameliorated by the NOX1 inhibitor ML171 (Zhang et al., 2022). Diabetic patients often exhibit increased blood glucose and oxidative stress, which enhance O-GlcNAcylation of multiple proteins through HBP. This contributes to mitochondrial dysfunction, endoplasmic reticulum stress, and metabolic disturbances, including those related to fatty acids and amino acids (Zhang et al., 2022). Recent studies have revealed that hyperglycemia increases O-GlcNAcylation of CaMKII, activating NOX2 and inducing oxidative stress, which can be suppressed by specific inhibitors (Lu et al., 2020). Additionally, Rac1/NOX2-mediated oxidative stress has been shown to induce apoptosis and exacerbate dysfunction in DCM (Kowluru and Kowluru, 2014). In various DCM models, NOX4 is overactivated, promoting oxidative stress and fibrosis through the TGF-β pathway and matrix metalloproteinases (Wang D. et al., 2023). Cardiac fibrosis and dysfunction are improved with the application of NOX4 antisense oligonucleotides in DCM (Maalouf et al., 2012). Furthermore, FOXO1 has been found to bind to the promoter of KLF5, thereby activating NOX4 and inducing oxidative stress, mitochondrial dysfunction, and impaired heart function (Kyriazis et al., 2021).

The development of DCM is closely linked to chronic hyperglycemia and insulin resistance, accompanied by systemic metabolic disorders, lipid accumulation, and advanced glycation end products. It is recognized that PPARα and PGC-1α are upregulated in early DCM but downregulated in advanced DCM, leading to reduced myocardial metabolic efficiency (Jia et al., 2016). Interestingly, the metabolic and pathological phenotypes observed in hearts with specific overexpression of PPARα are similar to those seen in DCM (Finck et al., 2002). Silencing PPARα in DCM has been shown to reduce NOX1 expression and oxidative stress, which in turn improves heart function (Wang L. et al., 2020). However, NOX-mediated oxidative stress increases when a PPARα activator is applied to macrophages, ultimately leading to ox-LDL production (Teissier et al., 2004). This suggests that PPARα-induced oxidative stress may exacerbate heart function in DCM by increasing FAO and decreasing energy efficiency. In contrast, recent studies have highlighted the cardioprotective role of PPARγ, leading to the use of several agonists to treat diabetes and its complications. Obesity and diabetes are accompanied by elevated inflammatory factors, such as TNF-α, which promote oxidative stress and reduce PPARγ expression (Su et al., 2013). The cardioprotective effects of the PPARγ activator rosiglitazone are likely due to its ability to reduce NOX4 expression (Guo et al., 2012). Additionally, rosiglitazone has been shown to activate AMPK, reducing oxidative stress mediated by NOX in endothelial cells exposed to high glucose levels (Ceolotto et al., 2007). Further research has revealed that silencing AMPK increases NOX2 expression, promoting apoptosis, pyroptosis, and ferroptosis in ischemia-reperfusion models under high glucose conditions (Wang C. et al., 2020). The glucagon-like peptide 1 (GLP-1) receptor agonist also protects the heart from high glucose toxicity by inhibiting p47phox translocation to the plasma membrane (Balteau et al., 2014).

The PPAR family and AMPK are key regulators of glycolipid and energy metabolism in DCM and play key roles in mitigating NOX-mediated oxidative stress. In HF, MI, and HCM, NOX4 has been recognized to maintain metabolic balance including glucose and lipid metabolism. However, NOX4 is currently only known to worsen DCM. Considering the metabolic characteristics of DCM, the role of NOX4 should not be summarized by producing oxidative stress alone, and further research is needed to demonstrate its regulation of metabolic function.

## 5 The crosstalk between NOX, mitochondrial dysfunction, and ferroptosis

Iron, an essential trace element, is present in the human body at an average amount of 3–4 g and participates in numerous processes, such as energy metabolism, essential for sustaining cell function. The redox properties of iron, including electron transfer, are crucial in forming Fe-sulfur clusters, hemoglobin, and other functional subunits (Ganz, 2013). Cardiomyocytes acquire iron through ferroportin (FPN), transferrin-bound iron via transferrin receptor 1 (TfR1), or non-transferrin-bound iron (NTBI) through various channels like L-type and T-type calcium channels, ZIP14 and DMT1 (Lakhal-Littleton et al., 2015). Both iron deficiency (ferritin <100 ng/mL or transferrin saturation <20%) and iron overload can damage cell function, underscoring the critical importance of maintaining intracellular iron homeostasis for cardiovascular health (Chung et al., 2023; Jankowska et al., 2013).

**FIGURE 5:**
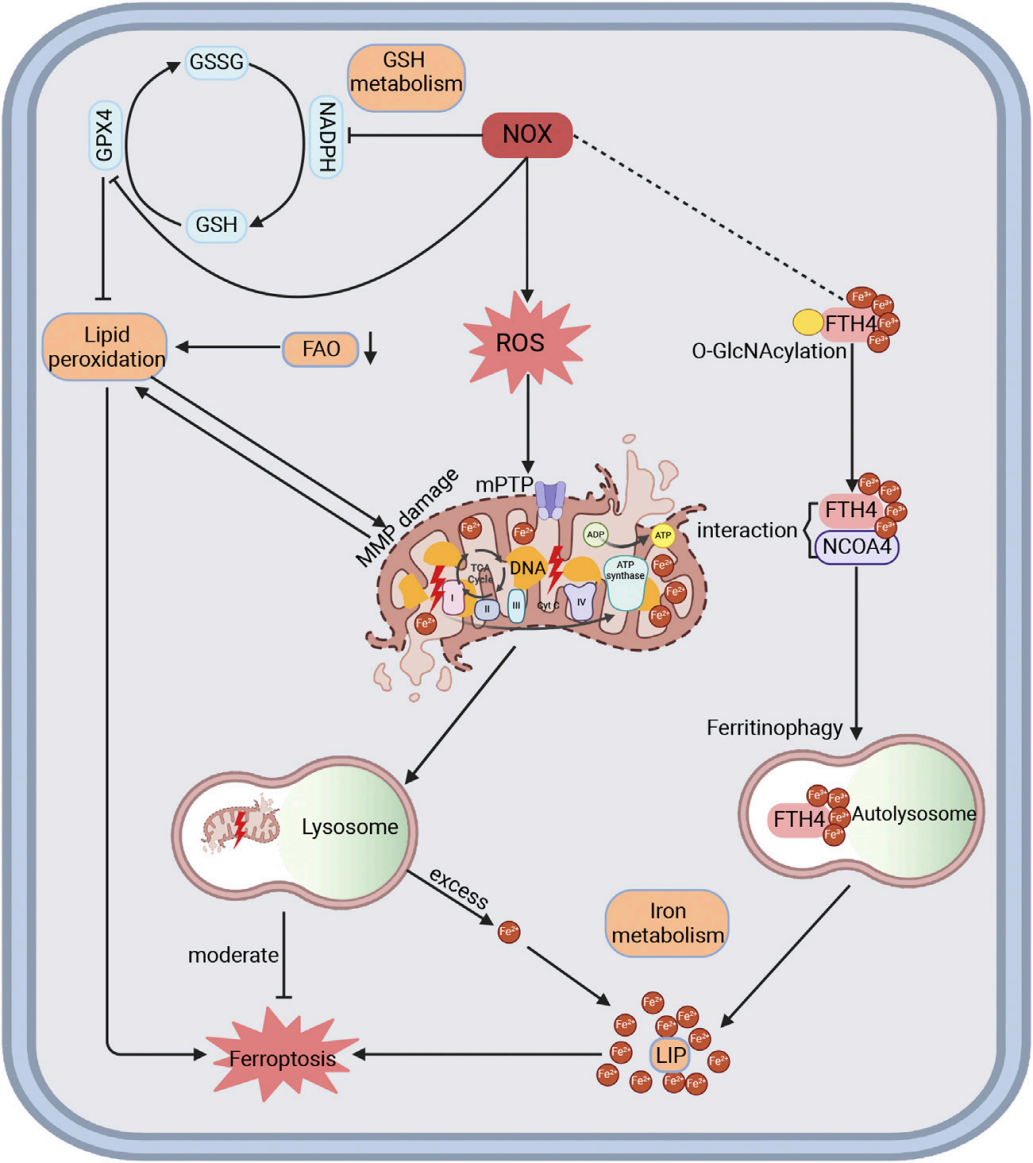
The interactions among NOX, mitochondria dysfunction, and ferroptosis in CVD. NOX is recognized to produce ROS and oxidative stress, leading to impaired membrane potential, DNA damage, electron transport chain damage, and lipid peroxidation of mitochondria, thereby increasing mitophagy in an attempt to restore mitochondrial function and mitigate ferroptosis in CVD. Excessive mitophagy can release Fe^2+^ into cytoplasm to form a labile iron pool and promote ferroptosis. Loss of O-GlcNAcylation in FTH could enhance its interaction with NCOA4, which induces ferritinophagy and expands the labile iron pool, thereby increasing ferroptosis. Here, NOX4 might affect the O-GlcNAcylation of target proteins to modulate ferroptosis. Moreover, NOX could also block GSH metabolism by consuming NADPH and inhibiting GPX4, thereby promoting lipid peroxidation and mitochondrial damage, which reduces protection against ferroptosis. Reduced FAO is demonstrated in HF, HCM, and ICM in proteomics, promoting lipid peroxidation and ferroptosis. FAO, fatty acid oxidation.

Mitochondrial dysfunction is regarded as a major contributor to intracellular oxidative stress, which significantly accelerates the progression of CVD (Ranjbarvaziri et al., 2021; Zhang et al., 2020). NOX-mediated oxidative stress induces mitochondrial dysfunction, myocardial remodeling, and cardiac dysfunction, which can be alleviated by NOX inhibitors and targeted mitochondrial antioxidants (Ago et al., 2010; Daiet al., 2011; Goh et al., 2019; Lozhkin et al., 2022). Ferroptosis, a form of programmed cell death regulated by lipid peroxidation and iron accumulation, is linked to disturbances in iron, lipid, and glutathione metabolism. Mitochondria, being iron-enriched organelles responsible for producing heme, Fe-S clusters, and ROS, exhibit morphological abnormalities and dysfunction, which contribute to ferroptosis (Chen et al., 2021; Fang et al., 2023). Additionally, NOX activation in CVD impedes the conversion of oxidized glutathione back to glutathione, reducing protection against lipid peroxidation damage (Fang et al., 2023). Excess ROS leads to mitochondrial DNA damage and a decrease in membrane potential, triggering lipid peroxidation, which enhances mitophagy in an effort to restore mitochondrial function and counteract ferroptosis (Su et al., 2023). However, excessive mitophagy releases abundant Fe^2^⁺ into the labile iron pool (LIP), inducing ferroptosis while increasing oxidative stress via the Fenton reaction and reducing ATP production (Yu et al., 2022). Previous studies have also shown that FAO decreases in CVD, leading to lipid accumulation and facilitating lipid peroxidation (Bertero and Maack, 2018; Chen et al., 2021; Previs et al., 2022; Ranjbarvaziri et al., 2021). Therefore, oxidative stress mediated by NOX not only diminishes protection against ferroptosis but also facilitates ferroptosis by inducing mitochondrial dysfunction and lipid peroxidation. Specifically, NOX2 has been shown to reduce the expression of the ferroptosis suppressor GPX4 in H9C2 cells exposed to high glucose and hypoxia/reoxygenation (Wang et al., 2020a). NOX4 impairs mitochondrial respiratory chain complexes I-V, leading to increased oxidative stress, mitochondrial fragmentation, and dysfunction, which in turn promotes iron accumulation and lipid peroxidation (Park et al., 2021). A recent study demonstrates that inhibiting O-GlcNAcylation leads to Fe^2^⁺ accumulation in mitochondria and increases mitophagy, while the loss of O-GlcNAcylation on ferritin heavy chain (FTH) increases interaction with nuclear receptor coactivator 4 (NCOA4), expanding the LIP and accelerating ferroptosis (Yu et al., 2022). Our research group has previously demonstrated that NCOA4-mediated ferritinophagy promote ferroptosis and myocardial fibrosis in DCM (Zhang et al., 2024). Interestingly, NOX4 has been shown to promote O-GlcNAcylation of CD36, increasing fatty acid uptake and providing novel insights into the regulation of mitochondrial dysfunction and ferroptosis (Nabeebaccus et al., 2017) (Figure 5).

## 6 NOX inhibitor

Current research on NOX inhibitors encompasses non-selective NOX inhibitors, selective NOX inhibitors, and specific peptide-based inhibitors. Among them, apocynin and diphenyleneiodonium chloride (DPI) are the most widely used non-selective NOX inhibitors, with off-target effects that broadly eliminated the production of ROS (Savla et al., 2021; Zhang et al., 2020). They are demonstrated to reduce oxidative stress mediated by NOX in MI, HCM, and DCM models (Cheng et al., 2021; Li et al., 2010; Saleem et al., 2018). Further advancements include non-selective NOX inhibitors such as VAS2870, VAS3947, ML171, and APX-115, which have shown efficacy in reducing oxidative stress mediated by NOX enzymes (Cha et al., 2017; Zhang et al., 2020). Apocynin, for example, has been shown to inhibit ROS production, potentially altering the protective effects of exercise during cardiac ischemia-reperfusion (Frasier et al., 2013). DPI, on the other hand, inhibits NOX4, which plays a role in promoting neovascularization through VEGF, impacting cardiac microcirculation and energy metabolism (Vogel et al., 2015). The dual nature of NOX4 in both protective and harmful roles in heart function underscores the need for selective NOX inhibitors. GKT137831, a selective NOX1/4 inhibitor, has demonstrated efficacy in reducing inflammatory macrophage infiltration, mitochondrial dysfunction, and cardiac remodeling in stress-induced cardiomyopathy and HCM models (Lozhkin et al., 2022; Vendrov et al., 2023; Zhao et al., 2015). Another dual inhibitor, GKT136901 (NOX1/4), has shown promise in cardiovascular diseases (Zhang et al., 2020). GSK2795039, the first NOX2 inhibitor, enhances macrophage efferocytosis and reduces apoptosis and 4-HNE production, potentially preventing plaque rupture in atherosclerosis (Wang Y. et al., 2023; Zhang et al., 2020). Similar to heart, the brain is an energy-sensitive organ that easily sufferes from ROS. NCATS-SM7270 is designed according to GSK2795039 to improve neuronal survival by suppressing NOX2. Interestingly, NOX4 knockdown in mild traumatic brain injury exacerbates cell death, which is partly reversed by NCATS-SM7270 (Mason et al., 2023). Selective NOX4 inhibitors such as GLX7013114 and GLX351322 have been identified for their protective effects against high glucose and palmitate-induced damage to islet cells (Anvari et al., 2015; Wang X. et al., 2018). Given the enigmatic role of NOX4 in CVD, the application of selective NOX4 inhibitors requires further validation. Meanwhile, peptide inhibitors targeting NOX1/2 have been designed and confirmed their effects (Altenhöfer et al., 2015) or instance, NoxA1ds has been shown to effectively reduce ROS production by suppressing NOX1 in models such as SHR (Camargo et al., 2018). Additionally, intranasal administration of NOX2ds-tat over 7 days has demonstrated improvement in trauma-associated olfactory deficits by inhibiting NOX2, a major contributor to oxidative stress in cardiovascular diseases (Liu et al., 2023).

## 7 Conclusion

During prolonged dietary alterations, systemic metabolic diseases, and pathological cardiac conditions, the limited metabolic reserve of the heart necessitates a metabolic substrate shift. This shift is often accompanied by increased oxidative stress, energy metabolism disorders, and the progression of CVD. This paper investigates the potential mechanisms by which NOX regulates energy metabolism, with a particular focus on the role of NOX4. In addition to its functions similar to the other NOX, NOX4 is also believed to regulate glucose, fatty acid and amino acid metabolism, thereby improving myocardial remodeling under stress challenges. Moreover, this study also preliminarily explores the mechanisms mediated by NOX to promote ferroptosis. NOX4 may regulate ferroptosis progression through the O-GlcNAcylation of proteins, potentially linking NOX4-mediated metabolic disorders with ferroptosis. Further investigation is warranted to substantiate these mechanisms.
